# Characterization of the *Aspergillus fumigatus* detoxification systems for reactive nitrogen intermediates and their impact on virulence

**DOI:** 10.3389/fmicb.2014.00469

**Published:** 2014-09-11

**Authors:** Katrin Lapp, Martin Vödisch, Kristin Kroll, Maria Strassburger, Olaf Kniemeyer, Thorsten Heinekamp, Axel A. Brakhage

**Affiliations:** ^1^Department of Molecular and Applied Microbiology, Leibniz Institute for Natural Product Research and Infection Biology – Hans Knöll Institute, JenaGermany; ^2^Department of Microbiology and Molecular Biology, Institute of Microbiology, Friedrich Schiller University Jena, JenaGermany; ^3^Integrated Research Treatment-Center – Center for Sepsis Control and Care, University Hospital Jena, JenaGermany

**Keywords:** *Aspergillus fumigatus*, reactive nitrogen intermediates, nitric oxide, virulence

## Abstract

*Aspergillus fumigatus* is a saprophytic mold that can cause life-threatening infections in immunocompromised patients. In the lung, inhaled conidia are confronted with immune effector cells that attack the fungus by various mechanisms such as phagocytosis, production of antimicrobial proteins or generation of reactive oxygen intermediates. Macrophages and neutrophils can also form nitric oxide (NO) and other reactive nitrogen intermediates (RNI) that potentially also contribute to killing of the fungus. However, fungi can produce several enzymes involved in RNI detoxification. Based on genome analysis of *A. fumigatus*, we identified two genes encoding flavohemoglobins, FhpA, and FhpB, which have been shown to convert NO to nitrate in other fungi, and a gene encoding *S*-nitrosoglutathione reductase GnoA reducing *S*-nitrosoglutathione to ammonium and glutathione disulphide. To elucidate the role of these enzymes in detoxification of RNI, single and double deletion mutants of FhpA, FhpB, and GnoA encoding genes were generated. The analysis of mutant strains using the NO donor DETA-NO indicated that FhpA and GnoA play the major role in defense against RNI. By generating fusions with the green fluorescence protein, we showed that both FhpA-eGFP and GnoA-eGFP were located in the cytoplasm of all *A. fumigatus* morphotypes, from conidia to hyphae, whereas FhpB-eGFP was localized in mitochondria. Because *fhpA* and *gnoA* mRNA was also detected in the lungs of infected mice, we investigated the role of these genes in fungal pathogenicity by using a murine infection model for invasive pulmonary aspergillosis. Remarkably, all mutant strains tested displayed wild-type pathogenicity, indicating that the ability to detoxify host-derived RNI is not essential for virulence of *A. fumigatus* in the applied mouse infection model. Consistently, no significant differences in killing of Δ*fhpA*, Δ*fhpB,* or Δ*gnoA* conidia by cells of the macrophage cell line MH-S were observed when compared to the wild type.

## INTRODUCTION

**FIGURE 1:**
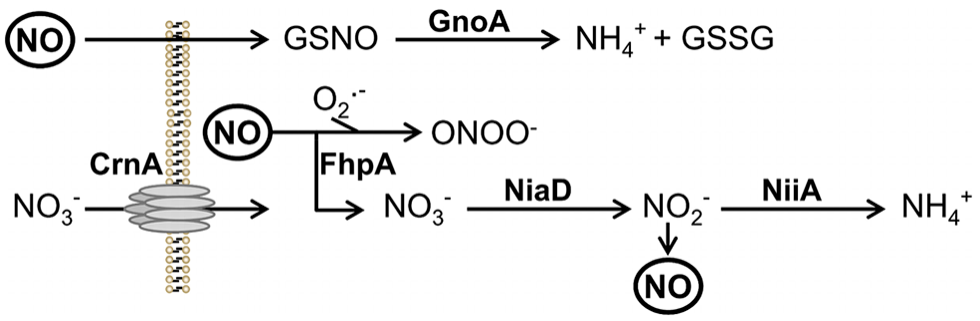
**Scheme of $NO_{3}^{-}$ reduction and NO detoxification.** Protein names are written in bold. Abbreviations: GSNO, nitrosylated glutathione; GSSG, reduced glutathione; ONOO^-^, peroxynitrite; $O_{2}^{•-}$, superoxide anion. NO, nitric oxide; NiaD, nitrate reductase; NiiA, nitrite reductase; CrnA, nitrate transporter.

The filamentous mold *Aspergillus fumigatus* is a ubiquitous saprophyte, degrading organic matter and thereby playing a key role in recycling a variety of carbon and nitrogen sources (Tekaia and Latgé, 2005). From a medical point of view *A. fumigatus* is also an important pathogen of humans (Brakhage, 2005). Diseases caused by *A. fumigatus* are wide-ranging, from allergies in immunocompetent hosts to systemic infections with invasive growth in patients with a severely weakened immune system, e.g., due to therapy of hematological malignancies, after stem cell or solid organ transplantation or suffering from chronic granulomatous disease. In immunocompromised patients, the lung is the major site of infection of *A. fumigatus*. The most prominent immune effector cells in the lung are alveolar macrophages and neutrophil granulocytes (Bogdan et al., 2000). Macrophages phagocyte resting spores and kill them mainly through oxygen dependent mechanisms (Philippe et al., 2003). Since contact with *A. fumigatus* hyphae induces an oxidative burst in neutrophil granulocytes, which is coupled to degranulation and the secretion of reactive oxygen intermediates (ROI) and also reactive nitrogen intermediates (RNI), an important role of these reactions in killing of *A. fumigatus* was suggested. However, deletion of the genes of two main regulators of oxidative stress response of *A. fumigatus*, the bZIP transcription factor *Af*Yap1 and the stress response regulator *Af*Skn7, did not affect virulence of the fungus in a mouse infection model (Lamarre et al., 2007; Lessing et al., 2007; Brakhage et al., 2010). Lambou et al. (2010), however, demonstrated that ROS detoxification by superoxide dismutases contributes to resistance against killing by murine macrophages but not to virulence. Since the importance of ROI for direct killing of *A. fumigatus* is therefore questionable, the role of RNI on the killing of *A. fumigatus* awaits further analysis. Nitric oxide (NO) is produced by the inducible NO synthase (iNOS) of host immune effector cells. After pathogen recognition, iNOS is stimulated by similar cytokines that are also required for induction of ROI production, e.g., IFN-γ, TNF-α, and IL-1 (Fang, 1997). It was shown that macrophages can produce up to 14 mM hydrogen peroxide and 57 μM NO (Brown et al., 2009). It is therefore likely that both radicals are present in infected tissue and that they interact to form highly reactive intermediates such as peroxynitrite (see **Figure 1**).

Microorganisms have evolved several protective mechanisms to counteract nitrosative stress. Reduced glutathione plays an essential role to survive nitrosative stress. At the same time, catalases and superoxide dismutases detoxify ROI and prevent formation of peroxynitrite. During moderate oxidative stress NO radicals are detoxified *via* nitrite or nitrate intermediates. In the pathogenic yeast *Candida albicans* the flavohemoglobin YHB1 was shown to be specifically involved in detoxification of NO radicals. Expression of *yhb1* was induced by contact of the fungus with macrophages (Chiranand et al., 2008). A *C. albicans yhb1Δ/yhb1Δ* mutant displayed moderately attenuated virulence in a murine infection model of disseminated candidiasis. However, the virulence defect of the *C. albicans yhb1Δ/yhb1Δ* mutant was not suppressed in mice defective for the NO synthase 2 (NOS2), indicating that not the reduced ability to detoxify RNI but another defect of the *yhb1Δ/yhb1Δ* mutant is the underlying cause for attenuation in virulence (Hromatka et al., 2005).

In *Cryptococcus neoformans* expression of glutathione reductase and thioredoxin peroxidase was induced by nitrosative stress. A glutathione reductase mutant was hypersensitive against NO and avirulent in a mouse infection model (Missall et al., 2006). The flavohemoglobin Fhb1 of *C. neoformans* catalyses the conversion of NO to nitrate. During nitrosative stress NO reacts with intracellular glutathione to *S*-nitrosoglutathione which is then reduced by the enzyme *S-*nitrosoglutathione reductase (Gno1) to form again oxidized glutathione (Fernández et al., 2003). Both genes, *gno1* and *fhb1*, are essential for virulence of *C. neoformans* (de Jesús-Berríos et al., 2003).

Here, we identified two flavohemoglobins, the cytosolic FhpA and the mitochondrial FhpB, and, in addition, the *S*-nitrosoglutathione reductase GnoA of *A. fumigatus*. We comprehensively analyzed the role of the respective genes *fhpA* (AFUA_4G03410), *fhpB* (AFUA_8G06080), and *gnoA* (AFUA_2G01040) with regard to detoxification of RNI and their impact on virulence.

## MATERIALS AND METHODS

### ETHICS STATEMENT

Mice were cared for in accordance with the principles outlined by the European Convention for the Protection of Vertebrate Animals Used for Experimental and Other Scientific Purposes (). All animal experiments were in compliance with the German animal protection law and were approved by the responsible Federal State authority “Thüringer Landesamt für Lebensmittelsicherheit und Verbraucherschutz” and ethics committee “Beratende Kommission nach §15 Abs. 1 Tierschutzgesetz” with the permit Reg.-Nr. 03-001/12.

### STRAINS, MEDIA AND CULTIVATION CONDITIONS

All *A. fumigatus* strains used in this study are listed in Table S1. *A. fumigatus* strain CEA17Δ*akuB* (da Silva Ferreira et al., 2006) was used to generate mutant strains Δ*fhpA*, Δ*fhpB*, Δ*gnoA*, Δ*gnoA/*Δ*fhpA*, Δ*gnoA/*Δ*fhpB*, and Δ*fhpA/*Δ*fhpB*. The strains were cultivated in *Aspergillus* minimal medium (AMM; Brakhage and Van den Brulle, 1995) containing 50 mM glucose and 70 mM NaNO_3_ as sole carbon and nitrogen source, respectively, if not otherwise stated. Alternative nitrogen sources were ammonium tartrate (20 mM) or glutamine (20 mM).

### STANDARD DNA TECHNIQUES, RNA EXTRACTION, cDNA SYNTHESIS, AND RT-PCR

Standard techniques for manipulation of DNA, including isolation of genomic *A. fumigatus* DNA and Southern blot analyses, were carried out as described earlier (Wartenberg et al., 2011). For RNA isolation and Northern blot analyses the protocols previously described (Bergmann et al., 2010) were followed. RNA samples were incubated with Turbo DNA-*free*^TM^ Kit (Ambion, USA) and transcribed in cDNA with the RevertAid^TM^ Premium First Strand cDNA Synthesis Kit according to the manufacturer’s protocol (Fermentas, Germany).

### GENERATION OF MUTANT STRAINS

Transformation of *A. fumigatus* protoplasts was performed as described earlier (Weidner et al., 1998). For deletion of *gnoA* (AFUA_2G01040) the flanking regions were amplified from genomic *A. fumigatus* DNA with the primer pairs gnoA_5for/gnoA_ptrA_5rev and gnoA_3rev /gnoA_ptrA_3for. The pyrithiamine resistance cassette *ptrA* was amplified with primers ptrA_forII/ptrA_revII from plasmid pSK275 (Szewczyk and Krappmann, 2010). For assembly of the *gnoA* deletion construct, a 3-fragment PCR according to the procedure described previously (Scharf et al., 2011) was performed with primers gnoA_5for and gnoA_3rev. For generating the *fhpB* (AFUA_8G06080) deletion mutant the flanking regions were amplified with the primers fhpB_5for/fhpB_hph_5rev and fhpB_3rev/fhpB_hph_3for. Here, hygromycin resistance was used as selection marker and the hygromycin resistance cassette was amplified from plasmid pUChph (Liebmann et al., 2004) with the primer pair hph_for/hph_rev. Assembly of the *fhpB* deletion construct was achieved by 3-fragment PCR with primers fhpB_5for/fhpB_3rev. The deletion construct to create the Δ*fhpA*-mutant was generated by amplifying the flanking regions of *fhpA* using the primer pairs fhpA_1(*Xma*I)/fhpA_2(*Sfi*I) and fhpA_3(*Sfi*I)/fhpA_4(*Xho*I). The resulting DNA fragments were separately cloned into pCR2.1TOPO (Invitrogen, Germany) and subsequently isolated by restriction with *Xma*I/*Sfi*I and *Sfi*I/*Xho*I. The pyrithiamine resistance cassette was obtained by *Sfi*I-restriction of plasmid pSK275. The *fhpA* DNA flanking fragments and the *Sfi*I-digested *ptrA* gene were cloned into plasmid p123 (Spellig et al., 1996) restricted with *Xma*I/*Xho*I. The resulting plasmid was digested with *Xma*I and *Xho*I and the deletion cassette was used to transform *A. fumigatus* CEA17Δ*akuB* or Δ*fhpB* to create the Δ*fhpA* mutant and the Δ*fhpA*/Δ*fhpB* double mutant, respectively. For creating the double mutants Δ*fhpA*/Δ*gnoA* and Δ*fhpB*/Δ*gnoA* the respective *gnoA* deletion cassettes conferring pyrithiamine or hygromycin resistance were employed to transform the mutants Δ*fhpA* and Δ*fhpB*, respectively. The *gnoA* deletion cassette containing the hygromycin resistance cassette was generated by 3-fragment PCR as described above employing primers gnoA_5for, gnoA_hph_5rev, gnoA_3rev, and gnoA_hph_3for. For complementation of the Δ*gnoA* mutant and construction of a GnoA-eGFP strain, the *gnoA* gene including 1 kb promoter sequence, was amplified from genomic *A. fumigatus* DNA using primers gnoA_Acc65I_for and gnoA_XmaI_rev. The DNA fragment was inserted in vector pJet1.2/blunt (Fermentas, Germany). The *gnoA* gene was inserted as *Acc*65I and *Xma*I fragment in plasmid pUCGH resulting in pUCGH_gnoA-egfp that was used to transform *A. fumigatus* Δ*gnoA*. Complementation of the Δ*fhpA* mutant by construction of an FhpA-eGFP strain is described in Kroll et al. (2014). For generation of strain FhpB-eGFP containing the *fhpB-egfp* gene fusion under control of the constitutive *otef* promoter, the *fhpB* gene was amplified by PCR using primers FhpB_XmaI_rev and FhpB_XmaI_for_otef. The DNA fragment was ligated *via Xma*I into plasmid pTH1 (a derivative of pUCGH carrying the pyrithiamine resistance cassette instead of the hygromycin resistance cassette) resulting in plasmid pTH-fhpB-egfp that was used for transformation of *A. fumigatus* CEA10. All PCR reactions were performed using Phusion high-fidelity DNA polymerase (Finnzymes, Finland). All oligonucleotides used in this study are listed in Table S2.

### INHIBITION ZONE ASSAY

Petri dishes with 10 ml bottom-agar were covered with a layer of 10 ml top-agar containing 1 × 10^8^ conidia of the respective strain. In the center, 100 μl of 45 mM DETA-NO (Sigma, Germany) were filled in a hole of 10 mm diameter.

### GERMINATION ASSAY

1 × 10^4^ conidia of *A. fumigatus* were cultivated in 50 μl AMM on cover slips and incubated at 37^∘^C in a wet chamber. The ratio of germinated to non-germinated conidia was determined by light microscopy by counting 100 conidia/germlings per time point and strain. The assay was repeated three times.

### FLUORESCENCE MICROSCOPY

Fifty microliter AMM were inoculated with 1 × 10^3^ conidia of strain FhpA-eGFP or GnoA-eGFP on glass cover slips and incubated at 37^∘^C. To analyze dormant conidia, conidia were inoculated in 50 μL H_2_O. For staining mitochondria, conidia of strain FhpB-eGFP were cultivated in 48 well plates with cover slips for 16 h in AMM. Fresh AMM with 500 nM Mitotracker Deep Red (Invitrogen) was added and staining was done for 30 min at 37^∘^C. Before fluorescence microscopy, the samples were washed three times with PBS. Samples were analyzed using a Leica DMI 4000B and a DM 4500B microscope (Leica Microsystems, Germany). Images were taken with a Leica DFC480 or a DFC 340FX camera and analyzed by Leica LAS V.3.7 software.

### KILLING ASSAY

Alveolar mouse macrophages (MH-S, ATCC CRL-2019) were grown in 12-well plates at a concentration of 7.5 × 10^5^ cells per well over night at 37^∘^C and 5% (v/v) CO_2_ in RPMI 1640 supplemented with 10% (v/v) FCS and 50 μg ml^-1^ gentamycin (PAA Laboratories, Germany). After washing with PBS the cells were co-incubated with 1.5 × 10^6^ *A. fumigatus* conidia for 20 min at 4^∘^C and then incubated for 6 h at 37^∘^C at 5% CO_2_. The samples were centrifuged with 5,000 *g* for 5 min and resuspended in 1 ml ice-cold water and incubated for 10 min on ice. The samples were mixed and diluted 1:1000 with PBS/0.01% (v/v) Tween 20. 100 μl of the sample were plated on Sabouraud agar plates and colonies were counted after incubation for 24 h at 37^∘^C. For determination of CFU, five technical replicates were analyzed.

### MOUSE INFECTION MODEL

Virulence of *A. fumigatus* mutant strains was tested in an established murine model for invasive pulmonary aspergillosis (Kupfahl et al., 2006; Schobel et al., 2010). Female CD-1 mice (Charles River, Germany) were immunosuppressed with cortisone acetate (25 mg per mouse intraperitoneally; Sigma-Aldrich, Germany) 3 days before and on the day of infection. Mice were anesthetized and intranasally infected with 20 μl of a fresh suspension containing 2 × 10^5^ conidia. A control group was mock-infected with PBS. To induce neutropenia in mice, cyclophosphamide (140 mg kg^-1^; Sigma–Aldrich, Germany) was injected intraperitoneally on days -4, -1, 2, 5, 8, and 11, with an additional subcutaneous dose of cortisone acetate (200 mg kg^-1^) on day -1. Mice were anesthetized and 3 × 10^4^ conidia in 20 μl PBS were applied intranasally.

The health status was monitored at least twice a day for 14 days and moribund animals, defined by severe dyspnoea, severe lethargy, or weight loss >20%, were sacrificed. Infections were performed with a group of 10 mice for each tested strain. Lungs from sacrificed animals were removed, and either stored in RNA*later* (Qiagen, Germany) for RNA extraction or fixed in formalin and paraffin-embedded for histopathological analyses according to standard protocols. RNA isolation and first-strand cDNA synthesis from infected lungs was performed as described previously (Schmaler-Ripcke et al., 2009).

### *Galleria mellonella* INFECTION MODEL

Virulence of the *A. fumigatus* Δ*fhpA*/Δ*fhpB* mutant was tested in a non vertebrate infection model using fresh larvae of the greater wax moth*Galleria mellonella* (Kavanagh and Reeves, 2004). For each strain 30 larvae (Quality Bugs, Linnich, Germany) were infected with 5 μl of an *A. fumigatus* spore suspension (5 × 10^7^ spores/ml in DPBS + 0.01% (v/v) Tween 20). Injection site was the lower right pseudopod. Larvae were checked for survival twice a day and accounted dead if nudging with forceps did not result in movement.

### STATISTICS

The Student’s *t*-test was used for significance testing of two groups. All significant differences are labeled with an asterisk (^∗^*p* ≤ 0.05; ^∗∗^*p* ≤ 0.01).

## RESULTS

### MOLECULAR CHARACTERIZATION OF THE *A. fumigatus* RNI DETOXIFICATION SYSTEM

To elucidate the role of RNI in the pathogenicity of *A. fumigatus*, genes encoding putative RNI-detoxifying enzymes were identified. BLAST analyses revealed that the *A. fumigatus* genome contains two putative orthologs to the yeast flavohemoglobin YHB1, named FhpA (AFUA_4G03410) and FhpB (AFUA_8G06080). The FhpA and FhpB amino acid sequence revealed 40 and 36% identity, respectively, to YHB1. FhpA and FhpB share 48% identical amino acids. As a third protein potentially involved in NO detoxification, GnoA (AFUA_2G01040) was identified as ortholog (73% identity) of the nitrosoglutathione reductase Gno1 previously characterized in *C. neoformans* (Zhou et al., 2009).

**FIGURE 2:**
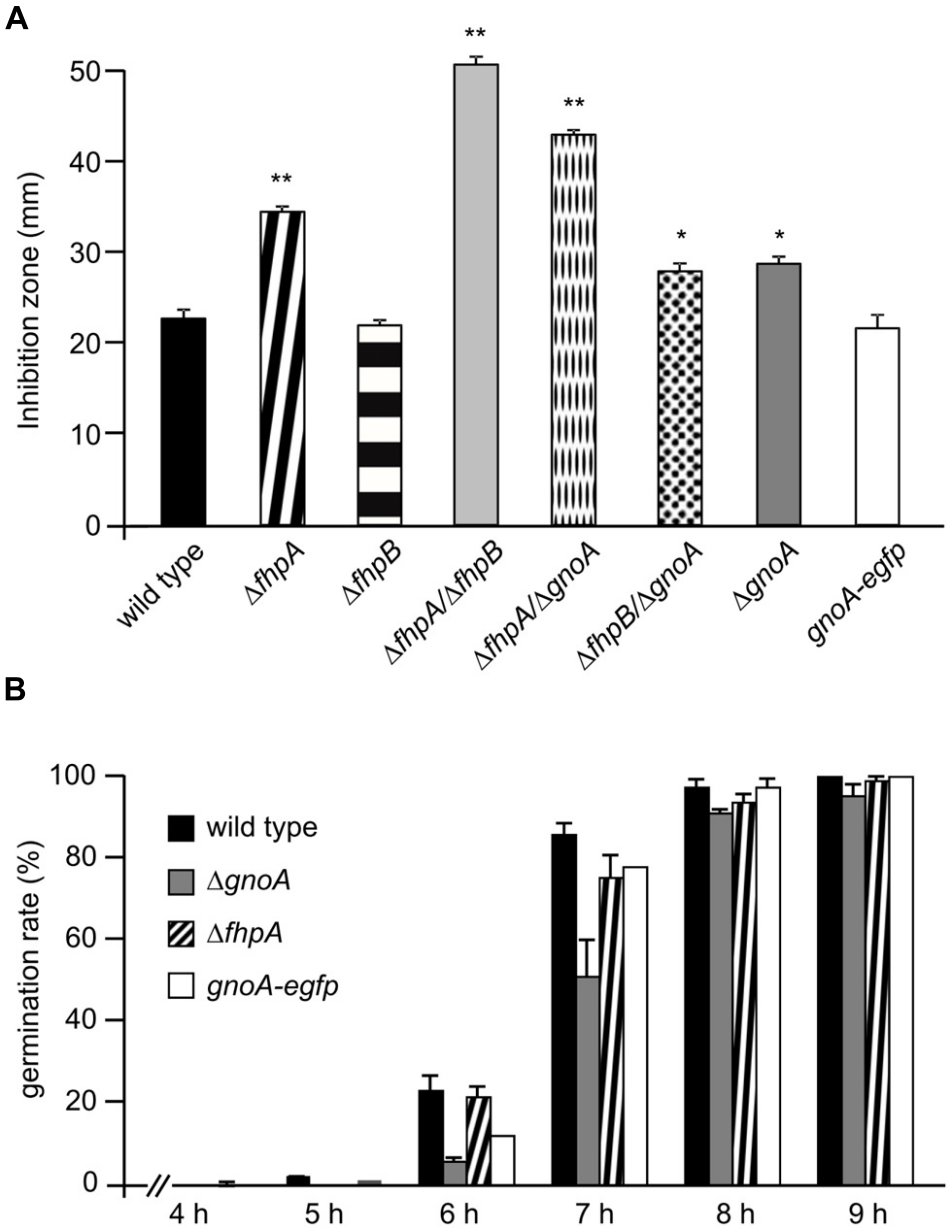
**Phenotypical characterization of mutant strains. (A)** Sensitivity of *A. fumigatus* strains against DETA-NO. The inhibition zones in a plate diffusion assay were measured 16 h after addition of 45 μM DETA-NO. *p-*values are indicated by asterisks; ^∗^*p* < 0.05; ^∗∗^*p* < 0.01. **(B)** Germination rate of the wild type and the Δ*fhpA* and Δ*gnoA* mutant strains. The ratio of germinated to non-germinated spores was calculated based on light microscopical inspection at the time points indicated.

**FIGURE 3:**
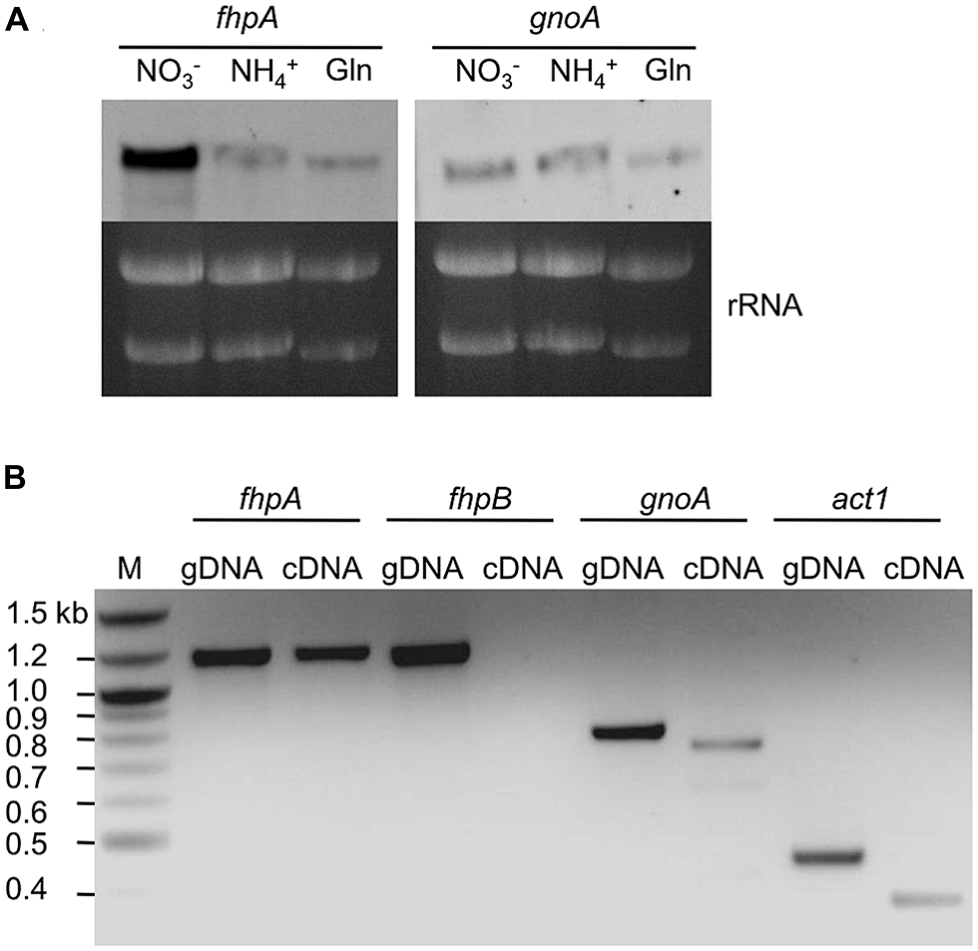
**Transcription of genes involved in RNI detoxification. (A)** Northern blot analysis of *fhpA* and *gnoA* mRNA steady state levels in *A. fumigatus* cultivated for 16 h in AMM with different nitrogen sources. Gln, glutamine; rRNA was used as loading control. **(B)** PCR on cDNA of lungs of mice infected with *A. fumigatus*. Immunosuppressed mice were infected with *A. fumigatus* wild-type conidia and sacrificed at day 5 post infection. Genomic DNA (gDNA) of *A. fumigatus* was used as control template and transcription of the gene encoding *A. fumigatus* actin (*act1*) was used as positive control.

Single and double deletion mutants of FhpA, FhpB, and GnoA encoding genes were generated (supplemental figures S1–S7). To test the sensitivity of *A. fumigatus* against nitrosative stress, an inhibition zone assay using DETA-NO as NO donor was performed (**Figure 2A**). Strains Δ*fhpA* and Δ*gnoA* showed increased sensitivity toward DETA-NO compared to the wild type or corresponding complemented strains *fhpA-egfp* and *gnoA-egfp*. Deletion of both flavohemoglobin-encoding genes *fhpA* and *fhpB* further increased RNI sensitivity. When grown on AMM agar plates with NaNO_3_ or NaNO_2_ as nitrogen source, all the mutants lacking the *gnoA* gene revealed a slight growth defect (data not shown), most likely due to the delay in germination of the Δ*gnoA* mutant (**Figure 2B**). The delay in germination was reduced when ammonium tartrate or glutamine were used as sole nitrogen source. Determination of the dry weight revealed that growth of all mutants was reduced in comparison to the wild type when grown in liquid medium with nitrate as sole nitrogen source. Replacing nitrate with glutamine complemented the growth defect in all flavohemoglobin mutants, except in the Δ*gnoA* mutant strain (data not shown). These results were supported by northern blot analyses monitoring mRNA steady state transcript levels of *fhpA* and *gnoA* (**Figure 3A**). In AMM with nitrate as sole nitrogen source, the *fhpA* gene was strongly expressed, indicating the need to detoxify RNI accumulating during nitrate metabolism. When the fungus was cultivated with ammonium tartrate or glutamine, transcription of *fhpA* was comparatively low. In contrast to *fhpA*, transcript levels of *gnoA* were similar in all media tested and were not affected by the nitrogen source. Transcripts of *fhpB* were not detected under these conditions (data not shown).

To proof whether genes encoding RNI detoxifying enzymes are involved in the infection process, their transcription was analyzed in lungs of infected mice. For this purpose, total RNA extracted from lungs of infected mice was isolated and transcription of genes was analyzed by RT-PCR (**Figure 3B**). As positive control, transcription of the *A. fumigatus act1* gene (actin; AFUA_6G04740) was monitored. The genes *fhpA* and *gnoA* were transcribed during the infection process, mRNA of *fhpB* was not detected.

### LOCALIZATION OF FhpA, FhpB, AND GnoA

**FIGURE 4:**
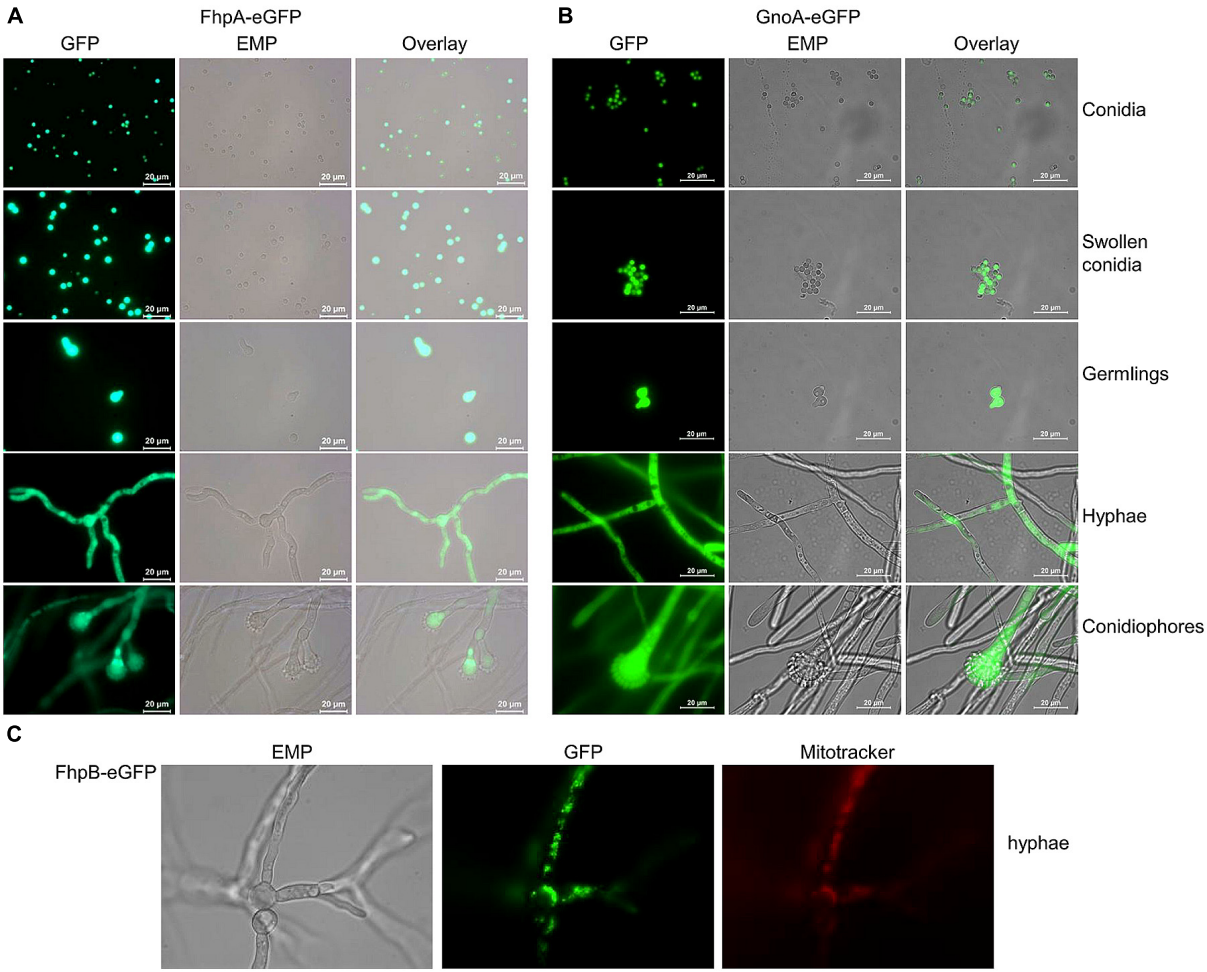
**Localization of FhpA-eGFP, GnoA-eGFP, and FhpB-eGFP using fluorescence microscopy. (A)** Fluorescence of strains FhpA-eGFP and **(B)** GnoA-eGFP was monitored in resting conidia (stored in H_2_O), in swollen conidia (incubation for 3 h in AMM), germlings (7 h AMM), hyphae (16 h AMM), and conidiophores (24 h AMM). **(C)** Fluorescence microscopy and staining with Mitotracker Red revealed localization of FhpB-eGFP in the mitochondria.

To determine the localization of FhpA and GnoA in *A. fumigatus*, the respective genes were fused to the *egfp* gene under control of their native promoter. Transformation of the Δ*fhpA* or the Δ*gnoA* strain with the *fhpA-egfp* or *gnoA-egfp* construct, respectively, resulted in transformants displaying wild-type phenotype, as tested by resistance to DETA-NO in agar plate diffusion assays (Figures S7 and S8). This data indicated that the respective fusion proteins were functional. Fluorescence microscopy revealed localization of both the FhpA-eGFP (**Figure 4A**) and the GnoA-eGFP protein (**Figure 4B**) in the cytoplasm of all *A. fumigatus* morphotypes, from conidia to hyphae. Due to low transcriptional activity of the *fhpB* promoter, fluorescence of a strain producing FhpB-eGFP was not detectable (data not shown). Therefore, a mutant was generated that produces FhpB-eGFP under control of the constitutive *otef* promoter (Figure S9). Fluorescence microscopy of strain FhpB-eGFP verified localization of this flavohemoglobin in the mitochondria (**Figure 4C**).

### CONFRONTATION OF MUTANT STRAINS WITH MACROPHAGES

To analyze whether the ability to detoxify macrophage-derived RNI influences fungal survival, the killing rate of wild type and mutant conidia confronted with macrophages was determined. No significant differences in killing of Δ*fhpA*, Δ*fhpB,* or Δ*gnoA* conidia by cells of the macrophage cell line MH-S were observed when compared to the wild type (Figure S10). This was also true for all double mutant strains deficient for RNI detoxification (data not shown).

### VIRULENCE OF RNI DETOXIFICATION MUTANTS

**FIGURE 5:**
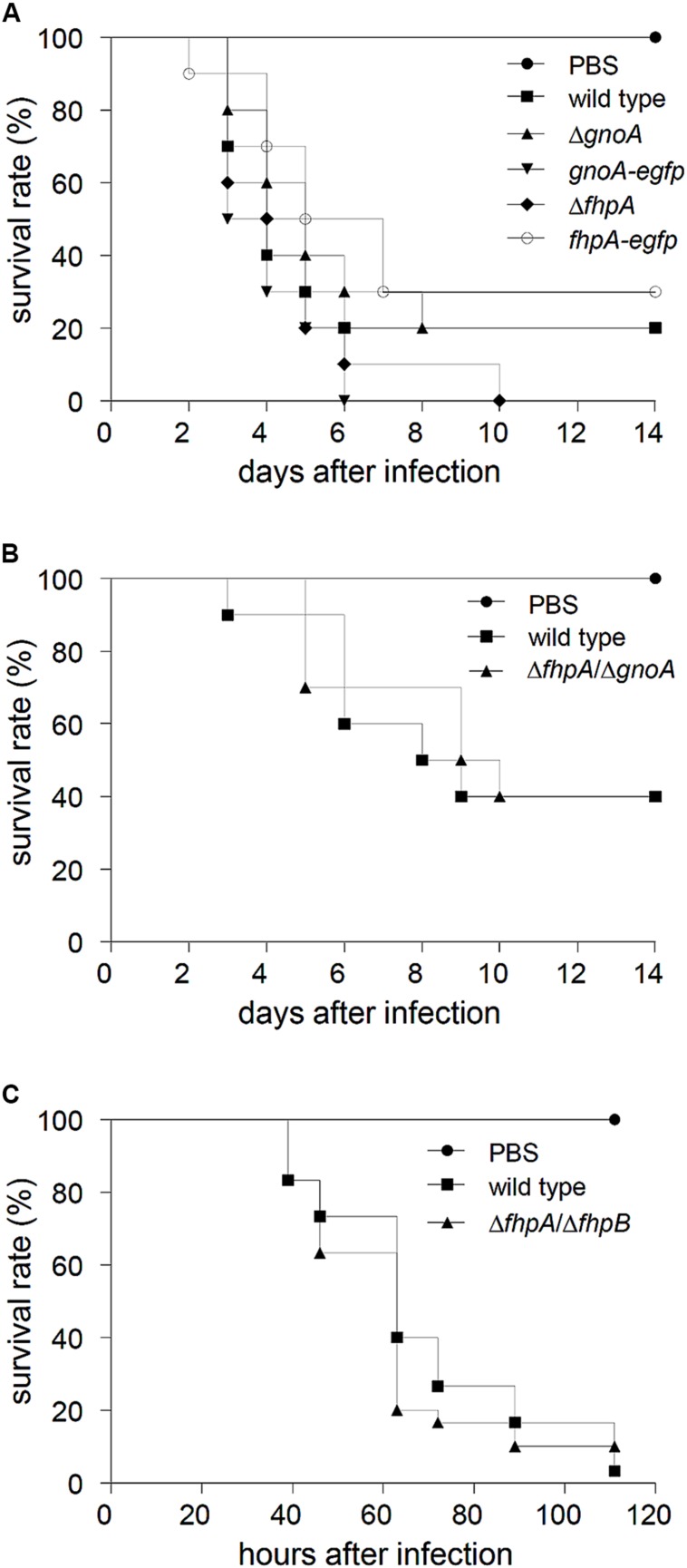
**Virulence studies. (A)** Cortisone acetate treated mice were infected with 2 × 10^5^ conidia of the indicated *A. fumigatus* strains. As control, a group of mice (*n* = 5) remained uninfected (inhalation of PBS). Survival was monitored for 14 days. **(B)** Neutropenic mice were infected with 3 × 10^4^ conidia of the indicated *A. fumigatus* strains. **(C)** Fresh larvae of the greater wax moth *G. mellonella* were infected with 5 × 10^5^ conidia of the wild type and the *ΔfhpA*/*ΔfhpB* mutant, respectively. As mock control PBS was used. Survival of larvae was monitored for 5 days.

The *gnoA* and *fhpA* mutant strains were tested in a mouse infection model for pulmonary aspergillosis. In this model, mice were immunosuppressed solely with cortisone acetate, i.e., neutrophils, that are able to produce NO, are still recruited to the site of infection. Survival of infected animals was monitored over a period of 14 days (**Figure 5A**). No significant differences in survival of mice infected with the mutant strains compared to mice infected with the wild type and the corresponding complemented strains occurred. Furthermore, histopathology of lungs of infected mice did not reveal any differences with regard to fungal growth or the recruitment of neutrophils (data not shown). In an alternative infection model, neutropenic mice, treated with cyclophosphamide, were infected with conidia of the Δ*fhpA*/Δ*gnoA* double deletion strain. Again, no difference in survival was detected compared to mice infected with wild-type conidia (**Figure 5B**). Additionally, virulence of the strain Δ*fhpA/*Δ*fhpB* was analyzed in a non-vertebrate model, using larvae of the greater wax moth *G. mellonella*. Also in this model, there was no difference in killing of larvae by the wild type or by the RNI detoxification mutants (**Figure 5C**).

## DISCUSSION

Immune cells eliminate pathogens by different mechanisms including the release of ROI during the oxidative burst and the production of RNI (Bogdan, 2011). The role of these reactive intermediates in defense against an infection with *A. fumigatus* is still a matter of debate. In a previous study that reported the impact of ROI and RNI on killing of *A. fumigatus* by alveolar macrophages, the authors speculated that ROI, but not RNI, are important in fungal killing (Philippe et al., 2003). Furthermore, triple deletion mutants of fungal superoxide dismutates showed a higher sensitivity against menadion and an impaired killing by alveolar macrophages (Lambou et al., 2010). However, more recent studies clearly showed that ROI are not directly involved in killing of *A. fumigatus* as ROI detoxification mutants of *A. fumigatus* are still fully virulent in mouse infection models (Lamarre et al., 2007; Lessing et al., 2007).

In *C. neoformans* two RNI detoxification systems exist (de Jesús-Berríos et al., 2003). The first system is represented by flavohemoglobins, which convert NO to nitrate *via* a nitrosyl intermediate. Similarly, as shown here, *A. fumigatus* harbors two flavohemoglobins, namely FhpA and FhpB. The resulting nitrate is metabolized by nitrate reductase NiaD to nitrite and then to ammonium *via* nitrite reductase NiiA (see **Figure 1**). The second system for RNI detoxification uses *S*-nitrosoglutathione (GSNO) reductases that reduce GSNO to ammonium and glutathione disulphide (GSSG). Here, GnoA was found to be involved in fungal germination. Testing the effect of different NO donors revealed that NO is able to negatively influence germination (Kunert, 1995). Therefore, the delayed germination in the mutants analyzed here, can be directly assigned to accumulation of RNI during initiation of germination. Consistently, all mutants with the exception of Δ*fhpB*, were more sensitive against NO when challenged with DETA-NO.

Screening fungal genome databases revealed that filamentous fungi like *Neurospora crassa*, *Gibberalla zeae* and also several *Aspergillus sp.* carry two putative genes encoding flavohemoglobins (te Biesebeke et al., 2010). In *A. fumigatus*, one of these genes, *fhpA*, seems to play a major role because it can compensate the lack of *fhpB*. This is in concordance with the results obtained from *A. nidulans* where mutants for *fhpA* but not *fhpB* grown on minimal medium with nitrite as sole nitrogen source showed drastically reduced growth (Schinko et al., 2010). Furthermore, similar to FhpA, the nitrate/nitrite reductase is localized in the cytoplasm, further supporting a predominant role of FhpA in RNI detoxification, whereas FhpB was localized in mitochondria. Consistently, in *A. oryzae* deletion of the *fhpB* homolog *fhp2* did not affect NO sensitivity. Transcript levels of *fhp2* were extremely low and in accordance with this observation fluorescence of an Fhp2-eGFP fusion protein was only detectable when an inducible promoter was employed. Fhp2 is located in the mitochondria and is putatively involved in detoxification of NO derived from nitrite metabolism (Zhou et al., 2011). In *A. fumigatus*, FhpB was recently found to be part of the proteome of resting conidia, indicating a function of this flavohemoglobin in the initial phase of stress response toward RNI (Teutschbein et al., 2010). This might also provide an explanation for the synergistic NO sensitivity of the *fhpA*/*fhpB* double mutant.

The function of flavohemoglobins seems to be specific for detoxification of RNI because neither Δ*fhpA* nor Δ*fhpB* showed altered sensitivity against oxidative stress-inducing agents like menadione, diamide, or H_2_O_2_ (data not shown). A similar result was found for the flavohemoglobin YHB1 of *S. cerevisiae*, because an *Sc*Δ*yhb1* mutant also exhibited specific activity restricted to RNI detoxification (Lewinska and Bartosz, 2006).

Similar to *A. nidulans*, *A. fumigatus* RNI detoxification mutants exhibited a growth defect when cultivated in AMM with nitrate as nitrogen source, which was rescued when the fungus was grown with other nitrogen sources like glutamine or ammonium tartrate. Metabolism of different nitrogen sources resulted in generation of different levels of RNI (Schinko et al., 2010), which was also reflected by different *fhpA* and *gnoA* mRNA steady state levels depending on the nitrogen source.

A first hint for a potential role of NO-detoxifying enzymes in virulence of *A. fumigatus* was obtained by detection of *fhpA* and *gnoA* transcripts in lungs of infected mice. Furthermore, Sugui et al. (2008) showed upregulation of *gnoA*-transcription in conidia confronted with neutrophils. In general, ROI, and RNI levels in the tissue increase during infection (Evans et al., 1993). RNI fulfill important functions in the defense against microorganisms. This was shown for bacteria (Chakravortty and Hensel, 2003), protozoa (Souza et al., 2010; Yeo et al., 2010) and also for human pathogenic fungi (Atochina et al., 2004; Hromatka et al., 2005; Nittler et al., 2005; Missall et al., 2006; Gonzalez et al., 2007). Miramon et al. (2012) showed that in *C. albicans* the *yhb1*Δ mutant is more susceptible to killing by neutrophils and wild-type sensitivity could be regained by addition of the NO scavenger carboxy-PTIO. Gross et al. (1999) reported that *A. fumigatus* enhanced the production of RNI in alveolar macrophages. However, as shown here, in co-incubation assays with macrophages of the MH-S cell line no differences in survival between NO detoxification-deficient mutants conidia and the wild-type conidia were detected. Certainly, the possibility remains that the level of RNI sensitivity in the mutant strains is not sufficient to exclude any impact of RNI detoxification on the interaction with immune cells. At least in the infection models applied in our study, deletion of genes involved in RNI detoxification did not alter virulence of *A. fumigatus*. To study *A. fumigatus* virulence in mice, two different models for immunosuppression are currently used. In the leukopenic model, animals are immunosuppressed by reiterate application of cyclophosphamide and a single low-dose of cortisone acetate, resulting in neutrophil depletion. In the second model immunosuppression is solely induced by administration of high doses of cortisone acetate, resulting in an impaired function of macrophages but still allowing recruitment of neutrophils. In our study, both murine infection models revealed wild-type pathogenicity of the RNI detoxification mutants. It may be the case that high levels of corticosteroid treatment impacts NO production capacity of immune cells. Therefore, in addition the *G. mellonella* infection model was employed. Haemocytes from *G. mellonella* are able to produce NO when they are confronted with pathogens or LPS (Krishnan et al., 2006; Semenova et al., 2014). Also in this invertebrate model that does not require immunosuppression, virulence of the mutants was not affected. Taken together, the *A. fumigatus* genome encodes two flavohemoglobins, FhpA and FhpB, and the *S*-nitrosoglutathione reductase GnoA, involved in RNI detoxification. FhpA and GnoA were found to represent the primarily responsible enzymes in *A. fumigatus* to counteract RNI stress. However, in different infection models RNI detoxification mutants revealed wild-type pathogenicity, implying that the ability to detoxify host-derived RNI is at least no major prerequisite for virulence of *A. fumigatus*.

## Conflict of Interest Statement

The authors declare that the research was conducted in the absence of any commercial or financial relationships that could be construed as a potential conflict of interest.
