# Supplementary material for: Characterization of the Aspergillus fumigatus detoxification systems for reactive nitrogen intermediates and their impact on virulence
Source: Front Microbiol. 2014 Sep 11;5:469. doi: 10.3389/fmicb.2014.00469 (PMC4160965; doi:10.3389/fmicb.2014.00469)
Supplement: Supplementary file 1 [file Presentation_1.ZIP › Supp Mat Table S1.PDF]

Table S1 *A. fumigatus* strains used in this study

Strain	Description	Source / reference
CEA10	wild type	CBS 144.89
Δ*akuB*	*akuB*^{KU80}::*pyrG*; *pyrG*$^{+}$, Δ*akuB* ^{KU80}	da Silva Ferreira *et al.*, 2006
Δ*fhpA*	*fhpA*::*ptrA*; Δ*fhpA*, PT^{R}	This study
Δ*fhpB*	*fhpB*::*hph*; Δ*fhpB*, Hyg^{R}	This study
Δ*fhpA*/Δ*fhpB*	*fhpA*::*ptrA*, *fhpB*::*hph*; Δ*fhpA*, Δ*fhpB*, Hyg^{R}, PT^{R}	This study
Δ*gnoA*	*gnoA*::*ptrA*; Δ*gnoA*, PT^{R}	This study
Δ*fhpA*/Δ*gnoA*	*fhpA*::*ptrA*, *gnoA*::*hph*; Δ*fhpA*, Δ*gnoA*, Hyg^{R}, PT^{R}	This study
Δ*fhpB*/Δ*gnoA*	*fhpB*::*hph*, *gnoA*::*ptrA*; Δ*fhpB*, Δ*gnoA*, Hyg^{R}, PT^{R}	This study
GnoA-eGFP = *gnoA*c	derived from Δ*gnoA*; contains *gnoA*-*egfp*-fusion construct under control of the *gnoA*-promoter; Hyg^{R}	This study
FhpA-eGFP = *fhpA*c	derived from Δ*fhpA*; contains *fhpA*-*egfp*-fusion construct under control of the *fhpA*-promoter; Hyg^{R}	Kroll *et al.*, 2014
FhpB-eGFP	contains *fhpB*-*egfp*-fusion construct under control of the *otef*-promoter; PT^{R}	This study

PT^{R}: Pyrithiamine-resistance; Hyg^{R}: Hygromycin-resistance
