# Supplementary material for: Characterization of the Aspergillus fumigatus detoxification systems for reactive nitrogen intermediates and their impact on virulence
Source: Front Microbiol. 2014 Sep 11;5:469. doi: 10.3389/fmicb.2014.00469 (PMC4160965; doi:10.3389/fmicb.2014.00469)
Supplement: Supplementary file 1 [file Presentation_1.ZIP › Supp Mat Table S2.PDF]

Table S2 Oligonucleotides used in this study

**Primer**	**Sequence 5‘→3‘**
gnoA_5for	GAAGCCGCAAAAGACAGCC
gnoA_3rev	CTCTCTTTATGCTCGATGC
gnoA_ptrA_5rev	GGCCTGAGTGGCCATCGAATTCCGAGTTCGGAGTTCGGATAG
gnoA_ptrA_3for	GAGGCCATCTAGGCCATCAAGCGTCGATATGTCGTAGGGGG
gnoA_hph_5rev	CGGATGATCAATAGTGCCACCGAGTTCGGAGTTCGGATAG
gnoA_hph_3for	GCGAATTCCAGCACACTGGCGTCGATATGTCGTAGGGGG
fhpB_5for	ACCCAGTACCTTCTAGATTGC
fhpB_hph_5rev	CGGATGATCAATAGTGCCACGGGAGAGTGATTTGAGCTGG
fhpB_hph_3for	GCGAATTCCAGCACACTGGCCCCACAGTCTTCTGAAATTCG
fhpB_3rev	GCGGAGAATCAAGGGAAAGG
ptrA_forII	GAATTCGATGGCCACTCAGGCC
ptrA_revII	GCTTGATGGCTTAGATGGCCTC
hph_for	GTGGCACTATTGATCATCCG
hph_rev	GCCAGTGTGCTGGAATTCGC
gnoA_Acc65I_for	GGTACCGAAGCCGCAAAAGACAGCC
gnoA_XmaI_rev	CCCGGGCATATCGACAACGCAGCGGA
fhpA_1(*Xma*I)	CACAAAGCCCGGGAAACC
fhpA_2(*Sfi*I)	TAGGGCCTGAGTGGCCGATGATGGCAACGCAGTCA
fhpA_3(*Sfi*I)	TAGGGCCATCTAGGCCGTACTACTACCGATTGCATTGC
fhpA_4(*Xho*I)	TAGCTCGAGGGTGGTATATTTCCCTCGGG
fhpA_5(*Hin*dIII)	AGCTAAGCTTCAACACCTTCTGGGGGTC
fhpA_6(*Bam*HI)	ACTAGGATCCATGAGGAACACCGCCAGTTC
FhpB_XmaI_for_otef	CCCGGGCCAGCTCAAATCACTCTCCC
FhpB_XmaI_rev	CCCGGGATCACGCGGAGCGCCAAAAAC
fhpB_for	CCTGTAACCCTACGGTCC
fhpB_rev	GGCCTTCATGAAAGGCTGC
