# Supplementary material for: Characterization of the Aspergillus fumigatus detoxification systems for reactive nitrogen intermediates and their impact on virulence
Source: Front Microbiol. 2014 Sep 11;5:469. doi: 10.3389/fmicb.2014.00469 (PMC4160965; doi:10.3389/fmicb.2014.00469)
Supplement: Supplementary file 1 [file Presentation_1.ZIP › Supp Mat Captions.pdf]

Supporting information

Table S1: *A. fumigatus* strains used in this study

Table S2: Oligonucleotides used in this study

Figure S1: Generation of strain Δ*fhpA*. Southern blot analysis to verify deletion of *fhpA*. Genomic DNA of *A. fumigatus* was digested with *Bam*HI. The probe was amplified by PCR using primers fhpA_1(*Xma*I) and fhpA_2(*Sfi*I) and hybridizes to the upstream region of *fhpA*. The expected size of the fragment for the wild type and the mutant is indicated.

Figure S2: Generation of strain Δ*fhpB*. Southern blot analysis to verify deletion of *fhpB*. Genomic DNA of *A. fumigatus* was digested with *Sac*I. The probe was amplified by PCR using primers fhpB_5for and fhpB_hph_5rev and hybridizes to the upstream region of *fhpB*. The expected size of the fragment for the wild type and the mutant is indicated.

Figure S3: Generation of strain Δ*gnoA*. Southern blot analysis to verify deletion of *gnoA*. Genomic DNA of *A. fumigatus* was digested with *Hind*III. The probe was amplified by PCR using primers gnoA_5for and gnoA_ptrA_5rev and hybridizes to the downstream region of *gnoA*. The expected size of the fragment for the wild type and the mutant is indicated.

Figure S4: Generation of strain Δ*fhpA*/Δ*gnoA*. Southern blot analysis to verify deletion of *gnoA* in strain Δ*fhpA*. Genomic DNA of *A. fumigatus* was digested with *Hind*III. The probe was amplified by PCR using primers gnoA_3rev and gnoA_hph_3for and hybridizes to the upstream region of *gnoA*. The expected size of the fragment for the wild type and the mutant is indicated.

Figure S5: Generation of strain Δ*fhpB*/Δ*gnoA*. Southern blot analysis to verify deletion of *gnoA* in strain Δ *fhpB*. Genomic DNA of *A. fumigatus* was digested with *Hind*III. The probe was amplified by PCR using primers gnoA_5for and gnoA_ptrA_5rev and hybridizes to the downstream region of *gnoA*. The expected size of the fragment for the wild type and the mutant is indicated.

Figure S6: Generation of strain Δ*fhpA*/Δ*fhpB*. Southern blot analysis to verify deletion of *fhpA* in strain *fhpB*. Probes were on the 5' flanking region of *fhpA* and *fhpB*. Genomic DNA of *A. fumigatus* was digested with *Bam*HI or *Sac*I. The probes were amplified by PCR using primers fhpA_1(*Xma*I) and fhpA_2(*Sfi*I) or fhpB_5for and fhpB_hph_5rev and hybridize to the downstream region of *gnoA*. The expected size of the fragment for the wild type and the mutants is indicated.

Figure S7: Generation of strain GnoA-eGFP. Southern blot analysis to verify integration of the *gnoA-egfp* construct in strain Δ*gnoA*. Genomic DNA of *A. fumigatus* was digested with *Hin*dIII. The probe was amplified by PCR using primers gnoA_5for and gnoA_ptrA_5rev and hybridizes to the downstream region of *gnoA*. The expected size of the fragment for the wild type and the mutants is indicated. Sensitivity towards DETA-NO was analyzed in an agar plate diffusion assay to verify functionality of the fusion protein.

Figure S8: Generation of strain FhpA-eGFP. Southern blot analysis to verify integration of an *fhpA-egfp* construct in the genome of strain Δ*fhpA*. Genomic DNA of *A. fumigatus* was digested with *Ssp*I. The probe was amplified by PCR using primers fhpA_1(*Xma*I) and fhpA_2(*Sfi*I) and hybridizes to the downstream region of *fhpA*. The expected size of the fragment for the wild type and the mutants is indicated. Sensitivity towards DETA-NO was analyzed in an agar plate diffusion assay to verify functionality of the fusion protein.

Figure S9: Generation of strain FhpB-eGFP. Southern blot analysis to verify integration of an *fhpB-egfp* construct under control of the otef promoter in the genome of strain CEA17. Genomic DNA of *A. fumigatus* was digested with *EcoR*I. The probe was amplified by PCR using primers fhpB_for and fhpB_rev and hybridizes to gene of *fhpB*. The expected size of the fragment for the wild type and the mutant is indicated. Fluorescence microscopy and staining with Mitotracker Red revealed localization of FhpB-eGFP in the mitochondria.

Figure S10: Killing assay. Survival of *A. fumigatus* wild type and RNI detoxification mutants was determined after co-incubation with mouse macrophages (MH-S).
