# Supplementary figures and images for: Characterization of the Aspergillus fumigatus detoxification systems for reactive nitrogen intermediates and their impact on virulence
Source: Front Microbiol. 2014 Sep 11;5:469. doi: 10.3389/fmicb.2014.00469 (PMC4160965; doi:10.3389/fmicb.2014.00469)

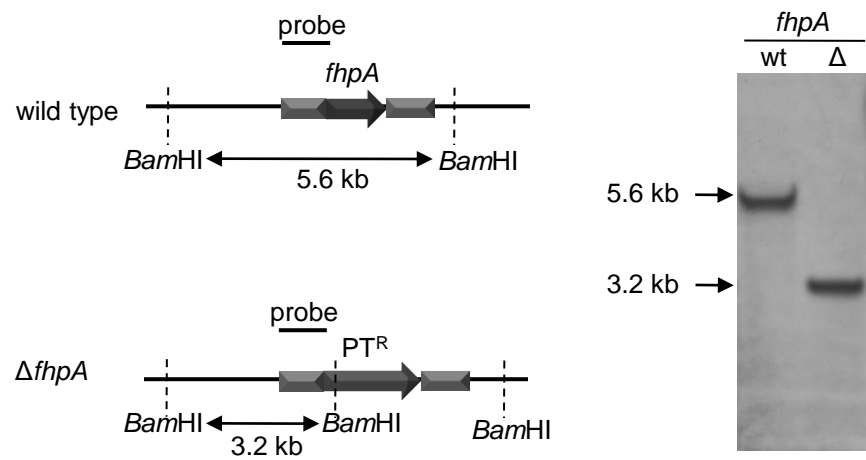

Fig. S1

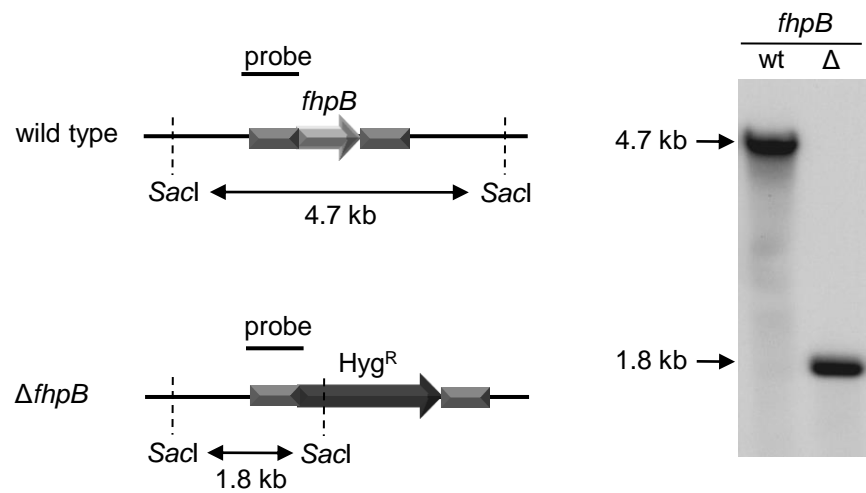

Fig. S2

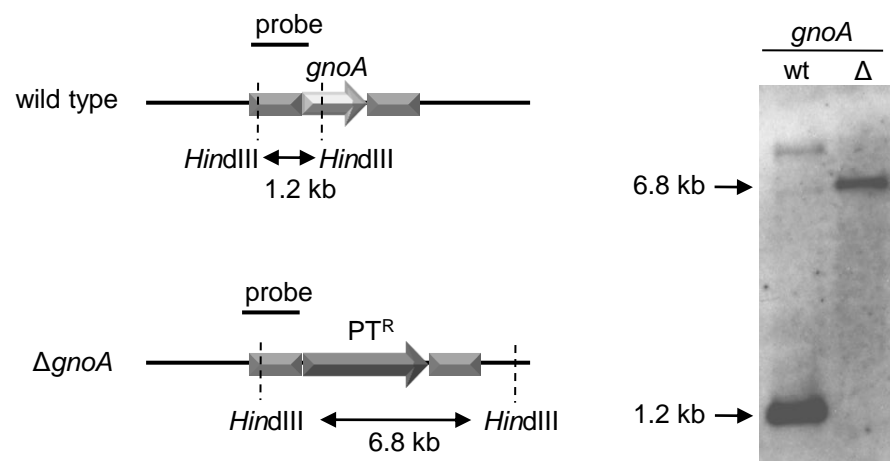

Fig. S3

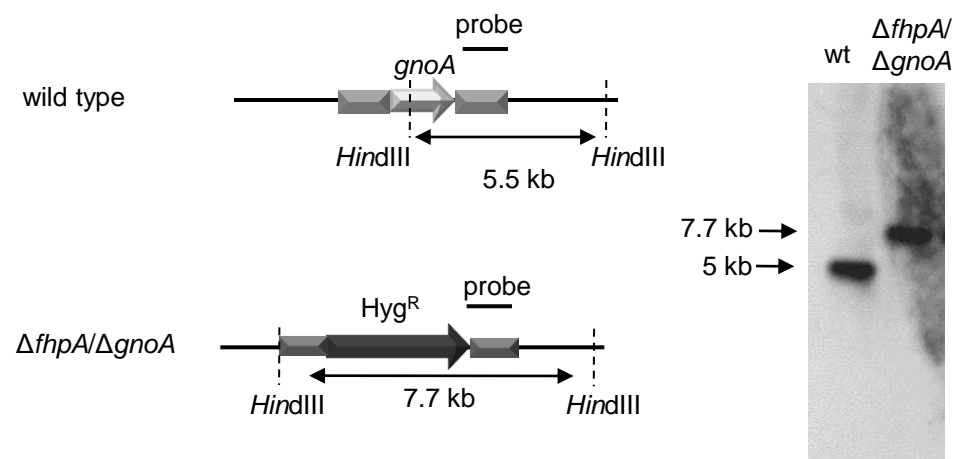

Fig. S4

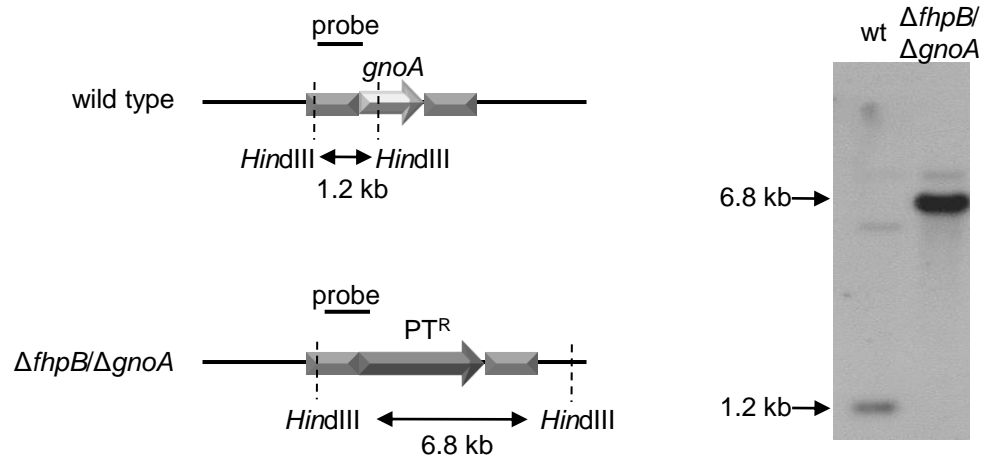

Fig. S5

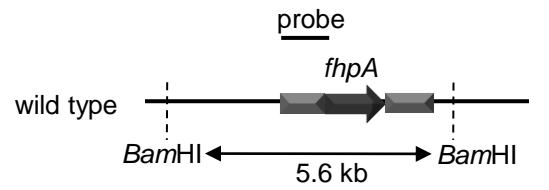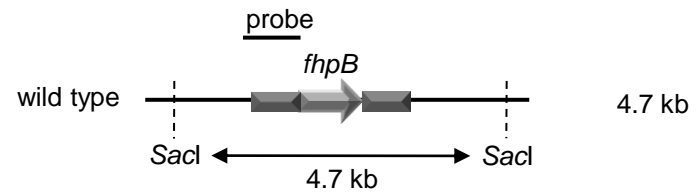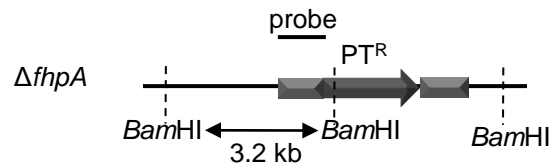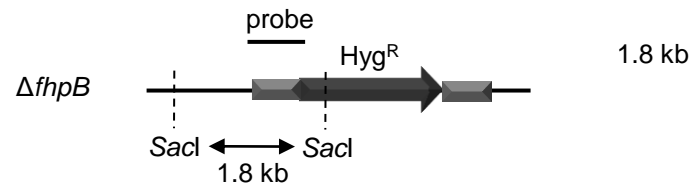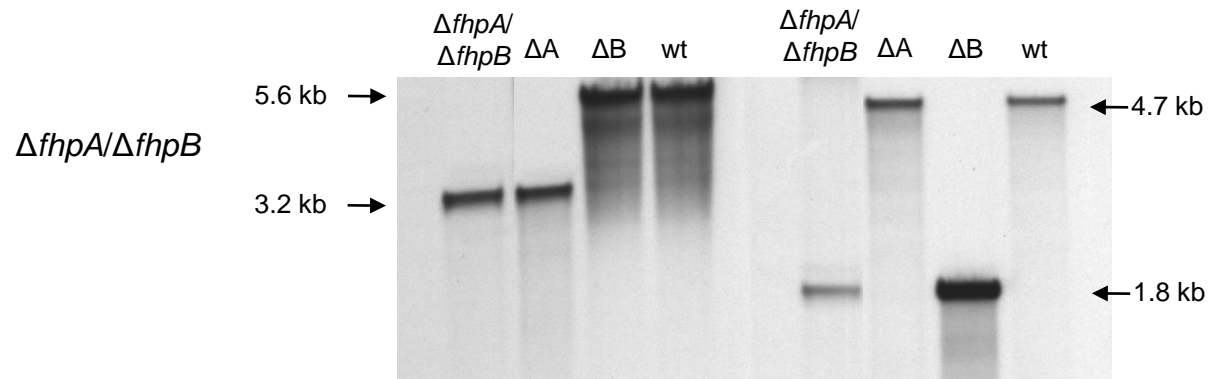

Fig. S6

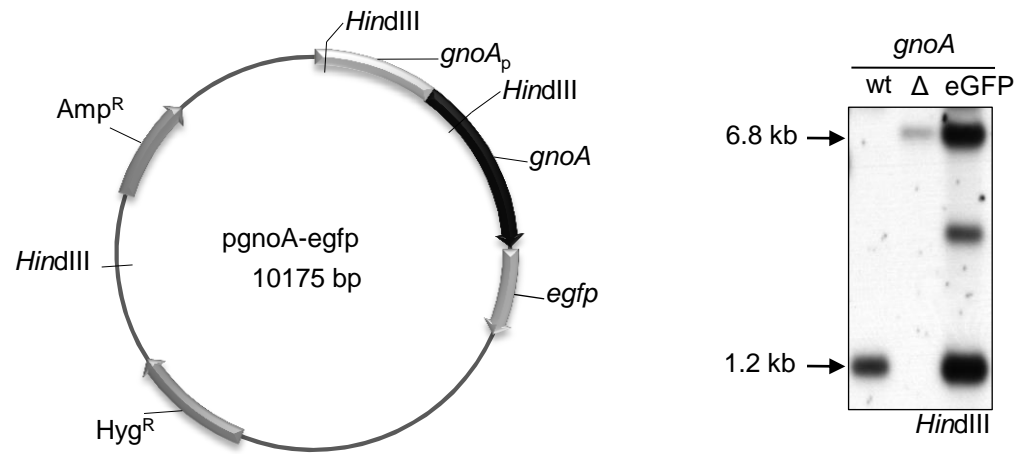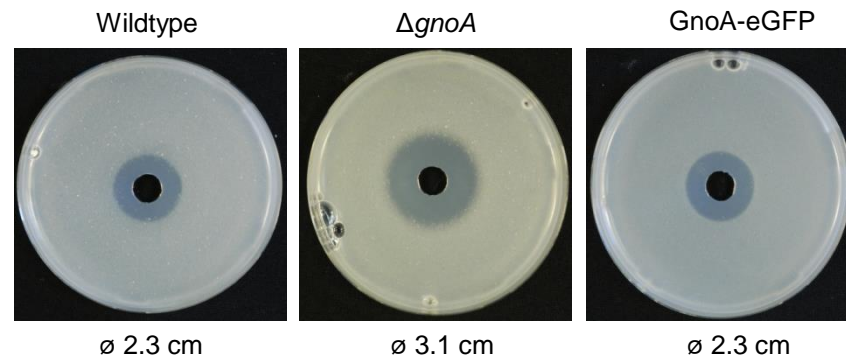

Fig. S7

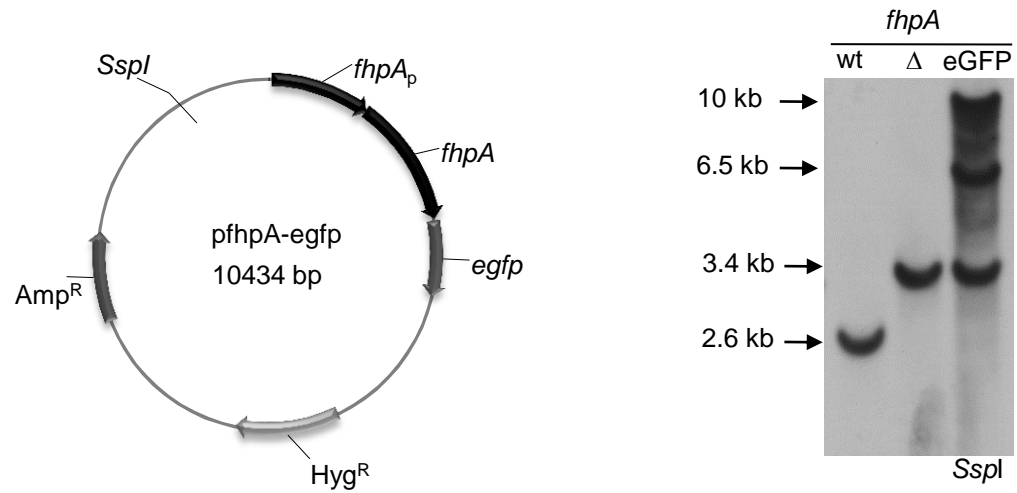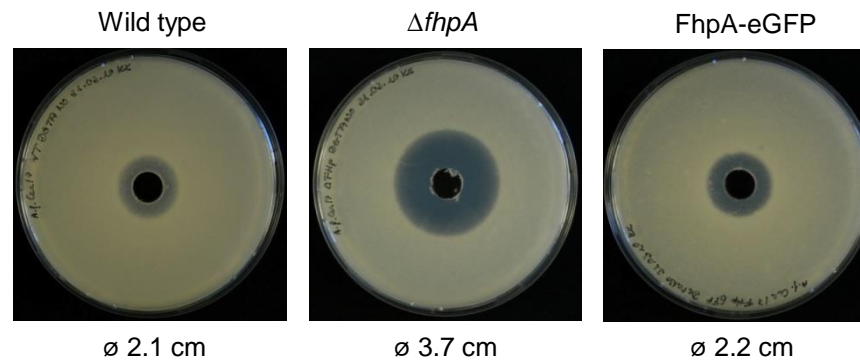

Fig. S8

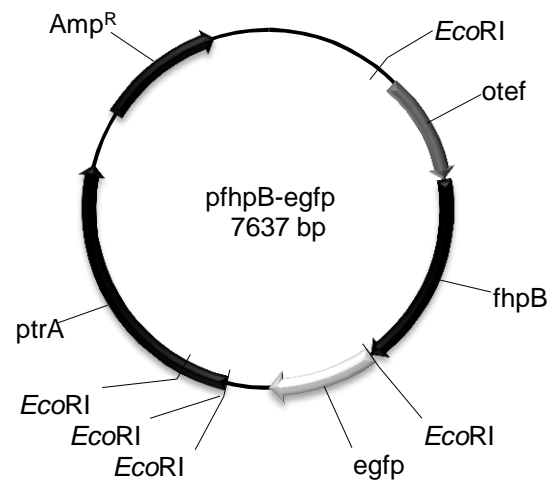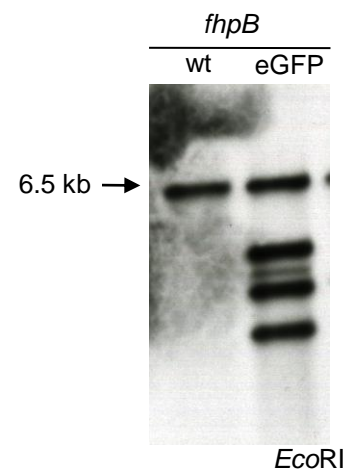

Fig. S9

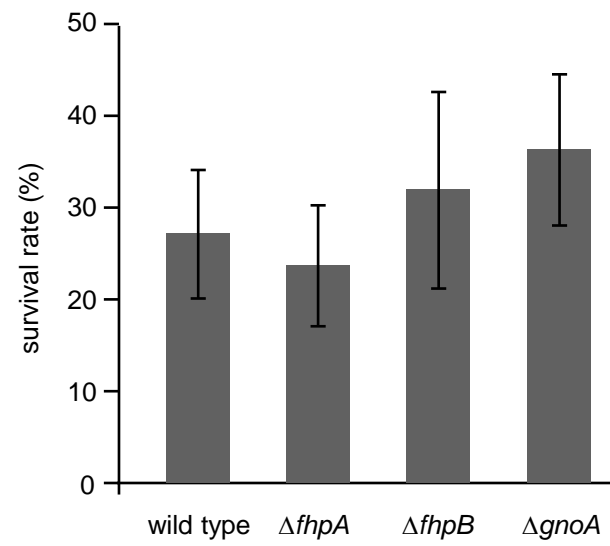

Fig. S10

Supplement: Supplementary file 1 [file Presentation_1.ZIP › Supp Mat Figures 1-10.PDF]
